# Prognostic factors for short‐term survival of cats that experienced postattenuation neurologic signs after surgical attenuation of single congenital portosystemic shunts

**DOI:** 10.1111/vsu.14267

**Published:** 2025-05-11

**Authors:** Ignacio Otero Balda, Laura E. Selmic, Polina Stamenova, Matthew Simpson, Victoria J. Lipscomb, Anne Kummeling, Nausikaa Devriendt, Hilde de Rooster, Katarzyna M. Grzywa, Michael S. Tivers, Guillaume Chanoit, Adrien Maggiar, Jean‐Philippe Billet, Román Soto Muñoz, Alberto Oramas, Ameet Singh, Ronan A. Mullins

**Affiliations:** ^1^ Department of Small Animal Surgery, Small Animal Clinical Studies Section, School of Veterinary Medicine, College of Health and Agricultural Sciences University College Dublin Dublin Ireland; ^2^ Department of Veterinary Clinical Sciences The Ohio State University Columbus Ohio USA; ^3^ Department of Clinical Science and Services Royal Veterinary College Hatfield UK; ^4^ Department of Clinical Sciences, Faculty of Veterinary Medicine Utrecht University Utrecht The Netherlands; ^5^ Small Animal Department, Faculty of Veterinary Medicine Ghent University Merelbeke Belgium; ^6^ Paragon Veterinary Referrals Paragon Business Village Wakefield UK; ^7^ Langford Vets Bristol UK; ^8^ Centre Hospitalier Vétérinaire Atlantia Nantes France; ^9^ Surgery Department Veterinary Specialists Ireland County Meath Ireland; ^10^ Department of Small Animal Clinical Sciences, College of Veterinary Medicine University of Florida Gainesville Florida USA; ^11^ Department of Clinical Studies, Ontario Veterinary College University of Guelph Guelph Ontario Canada

## Abstract

**Objective:**

To report 30‐day survival of cats that experienced postattenuation neurologic signs (PANS) after surgical attenuation of a single congenital portosystemic shunt (cPSS), and to investigate prognostic factors for short‐term survival.

**Study design:**

Multi‐institutional retrospective study.

**Sample population:**

A total of 59 cats with cPSS that experienced PANS.

**Methods:**

The medical records of 10 institutions were retrospectively reviewed to identify cats that underwent cPSS attenuation from January 1, 2010 through June 30, 2023 and developed PANS within 7 days postoperatively. Exclusion criteria were cats with arteriovenous malformation and cats lost‐to‐follow‐up prior to 30 days. Logistic regression identified factors associated with 30‐day survival. Odds Ratios (OR) and 95% CIs were calculated.

**Results:**

A total of 46 (78.0%) PANS‐affected cats survived to 30 days. A total of 13 (50.0%) of 26 cats that experienced postattenuation seizures (PAS) survived to 30 days, with most non‐surviving cats experiencing generalized PAS. Cats that experienced PAS (*p* < .01, OR: 0.015, 95% CI: <0.001–0.281) and treatment of PANS with propofol (*p* < .01, OR: 0.112, 95% CI: 0.022–0.569) were associated with decreased odds of 30‐day survival.

**Conclusion:**

Most cats that experienced PANS survived to 30 days; however, short‐term survival rate was worse for cats that experienced PAS.

**Clinical significance:**

The prognosis for cats that experience PANS is generally good but experiencing PAS and requiring treatment with propofol are negative prognostic factors.

## 1 INTRODUCTION

Development of postattenuation neurologic signs (PANS) is a potentially severe complication after surgical attenuation of congenital portosystemic shunts (cPSS) in cats.^1^, ^2^ Postattenuation neurologic signs include seizures but also milder signs such as blindness, ataxia, abnormal behavior, tremors, and twitching.^1^, ^2^ Strickland and colleagues^1^ defined PANS as occurrence of any neurologic signs between surgery and discharge, whereas others^3^ have defined postattenuation seizures (PAS) as those occurring within 7 days postoperatively. Postattenuation neurologic signs most commonly occurs within 96 h postoperatively.^1^, ^4^, ^5^, ^6^, ^7^, ^8^, ^9^, ^10^ A variety of terms have been used to describe this complication in cats including postsurgical seizures,^11^ postligation seizures,^4^ postligation neurologic dysfunction,^10^ postattenuation neurologic complications,^12^ and postattenuation neurologic signs.^1^, ^5^ The incidence of PANS in studies that include at least five cats is 14.3%–62.0%.^1^, ^4^, ^5^, ^6^, ^7^, ^8^, ^10^, ^12^, ^13^, ^14^ A recent meta‐analysis found the pooled prevalence of PANS in cats to be 38.9% (95% CI: 27–50.9), with the overall prevalence of PAS estimated to be 20.2% (95% CI: 15.3–25.1).^15^

Etiology of PANS is unknown; however, several theories have been proposed including changes in systemic and cerebrospinal fluid concentrations of endogenous benzodiazepines or imbalances in central nervous system neurotransmitter concentrations after cPSS attenuation; and perioperative disturbances in electrolyte, ammonia and glucose concentrations.^1^, ^2^, ^16^, ^17^ In cats, possible risk factors associated with PANS include lower grades of intraoperative postocclusion mesenteric portovenography and decreased osmolality 24 h postoperatively.^1^, ^2^, ^12^ Factors not shown to be associated with increased risk of PANS include cPSS morphology, signalment, method or degree of acute intraoperative cPSS attenuation, and presence of preoperative neurologic signs or seizures.^2^

Limited information exists regarding short‐term survival of PANS‐affected cats. To our knowledge, only three studies that include at least three PANS‐affected cats report 30‐day survival.^5^, ^14^, ^18^ Within such reports,^5^, ^14^, ^18^ which span over 20 years and include a total of only 28 PANS‐affected cats, the 30‐day survival rate is 66.7%–100.0%, and 50.0%–100.0% for cats with PAS. In dogs affected by PAS, having a history of preoperative seizures and development of focal PAS as opposed to generalized PAS are associated with improved short‐term survival.^3^, ^19^ However, no information exists regarding prognostic factors for short‐term survival of PANS‐affected cats. It is possible that with increased awareness of the occurrence of this condition in dogs and cats in more recent years combined with advancements in critical care medicine that improved survival would be observed, but this has not been investigated in cats. Therefore, the study objectives were to (1) report 30‐day survival of cats that experienced PANS after surgical attenuation of a single cPSS, and (2) investigate prognostic factors for short‐term survival. We hypothesized that (1) most PANS‐affected cats would survive to 30 days and (2) possible factors that would be positively associated with short‐term survival would include (i) development of PANS other than seizures, (ii) development of PANS in the second half of the study period, and (iii) for cats that experienced PAS, having a history of preoperative seizures.

## 2 MATERIALS AND METHODS

### 2.1 Inclusion and exclusion criteria

The medical records of 10 veterinary institutions were searched to identify cats that underwent surgical attenuation (suture ligation [SL], thin film banding [TFB], ameroid constrictor [AC]) of a single cPSS from January 1, 2010 to June 30, 2023 and developed PANS within 7 days postoperatively. Exclusion criteria included cats with arteriovenous malformation and those lost‐to‐follow‐up prior to 30 days. Ethical approval for the study was obtained from the University College Dublin research ethics committee (LS‐E‐21‐142‐Mullins). Postattenuation neurologic signs were defined as occurrence of any abnormal neurologic signs such as seizures, blindness, ataxia, tremors, abnormal behavior, twitching, and dullness that occurred within 7 days postoperatively.^2^, ^3^

### 2.2 Patient demographics

Data retrieved included breed, age, sex, neuter status, and bodyweight at the time of surgery.

### 2.3 Shunt morphology

Data retrieved included method of cPSS identification; cPSS morphology (single congenital intrahepatic portosystemic shunt [IHPSS] or single congenital extrahepatic portosystemic shunt [EHPSS] and submorphology) (left‐, central‐ or right‐divisional IHPSS, and portocaval or portoazygos EHPSS).

### 2.4 Preoperative neurologic signs

We collected data related to the presence and type of preoperative neurologic signs, including the presence of preoperative neurologic signs immediately prior to surgery.

### 2.5 Preoperative and immediate postoperative medical management, including pretreatment with levetiracetam

We gathered data regarding whether the cat received medical management (protein‐restricted/hypoallergenic diet, lactulose, and/or antimicrobials) for at least 1 week preoperatively, and whether it was continued immediately postoperatively. Concerning pretreatment with levetiracetam (LEV), cats were assigned as those that received no LEV (LEV‐); received LEV at ≥15 mg/kg every 8 h orally for ≥24 h preoperatively, ≥15 mg/kg every 8 h IV for ≥12 h preoperatively, or ≥ 40 mg/kg IV loading dose of LEV perioperatively, and continued at ≥15 mg/kg every 8 h for at least 5 days postoperatively (LEV1); or received LEV at <15 mg/kg, less than every 8 h frequency of administration, LEV started postoperatively only, or pre‐/intraoperative administration of LEV but without continuation postoperatively (LEV2).

### 2.6 Surgical details

Data retrieved included method and degree of acute intraoperative cPSS attenuation (complete/full, partial, or no attenuation), year of cPSS surgery, and number of cPSS surgeries performed.

### 2.7 PANS development and treatment of PANS

Data collected included timing and type (non‐seizure PANS, focal and generalized PAS) of PANS; details concerning treatment of PANS, and in particular drugs administered; occurrence of any severe complications during treatment of PANS (defined as those that could potentially affect survival such as aspiration pneumonia).

### 2.8 Serum electrolytes, ammonia, and glucose concentrations

Information regarding the most severe abnormality (increased or decreased) in electrolyte (sodium, potassium and chloride), glucose and ammonia concentrations within 7 days postoperatively but before PANS onset as well as postoperative values closest to PANS onset (within 24 h) was recorded.

### 2.9 Postoperative hospitalization and short‐term survival

Data recorded included duration of postoperative hospitalization for cats surviving to discharge; number of days from surgery to death/euthanasia for those not surviving to discharge; whether PANS remained at discharge; and whether the cat survived to 30 days postoperatively (defined as short‐term survival). For cats that underwent more than one cPSS attenuation surgery, 30‐day survival was defined as 30 days from the date of surgery after which the cat first experienced PANS. For cats that died/were euthanized prior to 30 days, cause of death or reason for euthanasia was recorded.

### 2.10 Statistical analysis

Continuous variables were tested for normality using graphical methods, skewness and kurtosis (values of 0 and 3, respectively), and Shapiro–Wilk tests. Univariable logistic regression analysis was performed to assess for factors associated with 30‐day survival. It was not feasible to perform a multivariable logistic regression analysis because to do so would require having at least 10 observations (cases) per independent variable evaluated, which was not possible in our study. OR and 95% CI were calculated. Factors assessed included breed, age, sex, neuter status, and bodyweight at surgery; presence of preoperative seizures; presence of neurologic signs immediately preoperatively, cPSS morphology and submorphology; method and degree of acute intraoperative cPSS attenuation; year of cPSS surgery and whether surgery took place in the first (January 1, 2010–December 31, 2016) or second (January 1, 2017–June 30, 2023) half of study period; whether the cat received medical management for 1 week preoperatively; LEV group; whether the cat received other anti‐seizure medication(s); whether the cat developed PAS; type of PAS; whether the cat received treatment for PANS; treatment of PANS with benzodiazepines, phenobarbital, LEV, propofol, and gabapentin; most severe sodium, potassium, chloride, glucose and ammonia concentrations within 7 days postoperatively; postoperative concentrations of sodium, potassium, chloride, glucose and ammonia closest to PANS onset; duration of postoperative hospitalization; and whether PANS remained at discharge. For the most severe electrolyte concentrations within 7 days postoperatively and postoperative values closest to PANS onset, heatmaps were created to illustrate the percentage of cases without derangements overall and among survivors and non‐survivors. Point biserial correlation was also performed to examine the relationship between selected dichotomous (sex [male vs. female], presence of preoperative seizures [yes vs. no], shunt morphology [EHPSS vs. IHPSS], degree of cPSS attenuation [partial or none vs. full], LEV group [LEV2 or LEV‐vs. LEV1], developed PAS [yes vs. no], treatment of PANS [yes vs. no], treatment of PANS specifically with propofol [yes vs. no], survival to discharge [yes vs. no] and survival to 30 days [yes vs. no]) and continuous (age [months] and bodyweight [kg] at surgery, timing of PANS development [hours postoperatively]) variables. Statistical analysis was performed using SAS Analytics Software. Statistical significance was set at *p* < .05.

## 3 RESULTS

A total of 59 PANS‐affected cats met the study inclusion criteria. Contributing institutions included Royal Veterinary College (26 cases), Utrecht University (9 cases), Ghent University (6 cases), Paragon Veterinary Referrals (6 cases), Langford Vets (4 cases), Center Hospitalier Vétérinaire Atlantia (3 cases), Veterinary Specialists Ireland (2 cases), University College Dublin (1 case), University of Florida (1 case), and University of Guelph (1 case).

### 3.1 Patient demographics

Median (range) age was 14 (4–55) months. The most common breeds were domestic shorthair, British shorthair and Ragdoll. Median (range) bodyweight was 2.9 (0.9–5.4) kg. There were 41 (69.5%) males and 18 (30.5%) females. A total of 33 (55.9%) cats were entire and 26 (44.1%) were neutered (Tables 1 and S1).

**TABLE 1:** Descriptive statistics of variables investigated for possible associated with 30‐days survival and cause of death for non‐survivors.

Patient	Breed	Age (months)	Sex	Weight (kg)	Preoperative neurologic signs	Neurologic signs immediately preoperatively	Preoperative seizures	Shunt morphology	Shunt subtype	Degree of shunt attenuation	Method of shunt attenuation
1	Ragdoll	4	FE	2.1	Yes	No	Yes	EHPSS	PC	Partial	TFB
2	BSH	4.5	ME	1.48	Yes	No	No	EHPSS	PC	Full	Full ligation
3	Ragdoll	5	ME	0.9	Yes	No	No	EHPSS	PA	Partial	Partial ligation
4	Ragdoll	5	FE	1.9	Yes	No	No	EHPSS	PC	Partial	TFB
5	Ragdoll	5	ME	1.85	Yes	No	No	EHPSS	PC	Partial	TFB
s6	BSH	5.3	FE	2.2	Yes	No	No	EHPSS	PC	Partial	Partial ligation
7	BSH	5.6	ME	2.4	Yes	No	No	EHPSS	PC	None	ARC
8	DSH	5.8	ME	3.5	Yes	No	Yes	IHPSS	LD	Partial	TFB
9	Ragdoll	5.8	ME	5.4	Yes	No	No	EHPSS	PC	Partial	ARC
10	DSH	6	ME	1.65	Yes	No	Yes	EHPSS	PA	Partial	Partial ligation
11	BSH	6	MN	2.4	Yes	No	No	EHPSS	PC	Full	Full ligation
12	BSH	6	ME	1.75	Yes	No	No	IHPSS	LD	Partial	Partial ligation
13	DSH	6	FE	1.65	Yes	No	Yes	EHPSS	PC	Partial	Partial ligation
14	British Blue	6	ME	1.5	Yes	No	No	EHPSS	PC	Partial	TFB
15	Maine Coon	6	MN	3.5	Yes	No	No	EHPSS	PC	Partial	TFB
16	Ragdoll	6	ME	3.2	Yes	No	No	EHPSS	PC	Partial	TFB
17	BSH	6	ME	2.4	Yes	No	No	EHPSS	PC	Partial	TFB
18	DSH	6.7	ME	1.5	Yes	No	No	EHPSS	PC	Partial	Partial ligation
19	BSH	7	ME	2.19	Yes	No	No	EHPSS	PC	Partial	TFB
20	DSH	7	MN	2.6	Yes	No	Yes	EHPSS	PC	Full	Full ligation
21	DSH	7	ME	2.62	Yes	No	No	EHPSS	PC	Partial	Partial ligation
22	Ragdoll	7	ME	2.2	Yes	No	No	EHPSS	PC	Partial	TFB
23	Birman	7	ME	2.7	Yes	No	No	EHPSS	PC	Partial	TFB
24	Ragdoll	7	FN	2.2	Yes	No	No	EHPSS	PC	Partial	TFB
25	DSH	7	FN	2.43	Yes	No	No	EHPSS	PC	Partial	Partial ligation
26	BSH	7	ME	2.57	Yes	No	No	IHPSS	LD	Partial	Partial ligation
27	DSH	7	ME	2.6	Yes	No	No	EHPSS	PC	Partial	Partial ligation
28	Maine Coon	8	FN	3.05	Yes	No	No	EHPSS	PA	Partial	TFB
29	DSH	8	ME	2.6	Yes	No	No	EHPSS	PC	Partial	TFB
30	Ragdoll	8.1	ME	3.4	Yes	No	No	EHPSS	PC	Partial	Partial ligation
31	Maine Coon	8.5	ME	2.7	Yes	No	No	EHPSS	PC	Partial	Partial ligation
32	BSH	8.9	FE	2.45	Yes	No	No	EHPSS	PC	Partial	ARC
33	DSH	9	FE	3.9	Yes	No	Yes	EHPSS	PC	Full	Full ligation
34	DSH	9	FN	3.03	Yes	No	No	EHPSS	PA	Partial	ARC
35	Ragdoll	9	MN	2.5	Yes	Yes	Yes	EHPSS	PC	None	TFB
36	DSH	9	ME	2.95	Yes	No	No	EHPSS	PC	None	Partial ligation
37	BSH	11	MN	3.8	Yes	No	Yes	EHPSS	PC	Full	Full ligation
38	DSH	10	FN	2.65	Yes	No	No	EHPSS	PC	Partial	ARC
39	DSH	11	MN	3.49	Yes	Yes	No	EHPSS	PC	Partial	TFB
40	Savannah	12	MN	3.0	Yes	No	Yes	EHPSS	PC	None	ARC
41	Bengal	12	ME	4.6	Yes	No	No	EHPSS	PC	Partial	ARC
42	Persian	12	FN	2.62	Yes	No	No	EHPSS	PC	Partial	TFB
43	DSH	12	ME	0.9	Yes	No	No	EHPSS	PC	Partial	TFB
44	DSH	12	FE	2.0	Yes	No	No	EHPSS	PC	Full	Full ligation
45	DSH	14	MN	3.25	Yes	No	No	EHPSS	PC	Full	Full ligation
46	BSH	16	FN	4.5	Yes	No	No	EHPSS	PC	Partial	ARC
47	DSH	16	MN	3.3	Yes	No	No	EHPSS	PC	Full	Full ligation
48	Bengal	17	MN	3.9	Yes	No	No	EHPSS	PC	Full	Full ligation
49	Ragdoll	27	MN	3.85	Yes	No	Yes	EHPSS	PC	Partial	Partial ligation
50	Selkirk Rex	33	MN	2.8	Yes	No	No	EHPSS	PC	Partial	TFB
51	DSH	29	MN	4.3	Yes	No	No	EHPSS	PC	Partial	ARC
52	DSH	29	MN	4.1	Yes	No	Yes	EHPSS	PC	Full	Full ligation
53	DLH	35	MN	3.25	Yes	No	Yes	EHPSS	PC	Partial	Partial ligation
54	DSH	31	FE	4.6	Yes	No	No	EHPSS	PC	Partial	TFB
55	DSH	40	FN	3.4	Yes	No	No	IHPSS	LD	Full	Full ligation
56	DSH	50	MN	3.8	Yes	No	No	EHPSS	PC	Partial	TFB
57	BLH	51	FN	5.3	Yes	No	No	EHPSS	PC	Full	Full ligation
58	DSH	51	ME	2.7	Yes	No	Yes	EHPSS	PC	Full	Full ligation
59	DSH	55	FN	3.05	Yes	No	No	EHPSS	PC	None	ARC

Patient	Number of shunt surgeries	LEV group	Developed PAS	Type of PAS	Timing of development of PANS (hours)	Treatment of PANS	Treatment of PANS with propofol	Survival to discharge	PANS remaining at discharge	Survival to 30 days postoperatively	Cause of death
1	1	LEV2	No		72	Yes	No	Yes	No	Yes	
2	1	LEV‐	Yes	Generalized	16	Yes	Yes	No		No	PANS related
3	1	LEV‐	Yes	Generalized	2	Yes	No	No		No	Non‐PANS related
4	1	LEV1	No		6	Yes	No	Yes	Yes	Yes	
5	1	LEV2	Yes	Unknown	0.5	Yes	No	No		No	PANS related
6	1	LEV‐	No		5	No	No	Yes	No	Yes	
7	1	LEV‐	No		60	No	No	Yes	Yes	Yes	
8	1	LEV‐	Yes	Generalized	42	Yes	No	No		No	PANS related
9	1	LEV‐	No		7	No	No	Yes	Yes	Yes	
10	1	LEV2	Yes	Generalized	12	Yes	No	Yes	Yes	Yes	
11	1	LEV1	Yes	Unknown	48	Yes	No	Yes	Yes	Yes	
12	2	LEV‐	No		12	Yes	Yes	Yes	Yes	Yes	
13	2	LEV1	No		6	Yes	No	Yes	Yes	Yes	
14	1	LEV‐	Yes	Generalized	24	Yes	Yes	No		No	PANS related
15	2	LEV2	No		12	No	No	Yes	No	Yes	
16	1	LEV‐	Yes	Unknown	48	Unknown	Unknown	No		No	PANS related
17	1	LEV1	No		48	Yes	No	Yes	Yes	Yes	
18	1	LEV‐	No		0.5	Unknown	Unknown	Yes	Yes	Yes	
19	1	LEV‐	No		0.5	Yes	No	Yes	Yes	Yes	
20	1	LEV1	No		12	Yes	No	Yes	No	Yes	
21	2	LEV2	Yes	Partial	24	Yes	No	Yes	Yes	Yes	
22	1	LEV1	Yes	Generalized	24	Yes	No	Yes	Yes	Yes	
23	1	LEV‐	Yes	Unknown	68	Yes	Yes	Yes	Yes	Yes	
24	1	LEV1	Yes	Generalized	18	Yes	No	Yes	Yes	Yes	
25	1	LEV1	No		72	Yes	No	Yes	No	Yes	
26	1	LEV‐	Yes	Generalized	24	Yes	No	No		No	PANS related
27	1	LEV‐	No		12	Yes	No	Yes	Yes	Yes	
28	1	LEV‐	No		0.5	Yes	No	Yes	No	Yes	
29	1	LEV2	Yes	Unknown	24	Yes	Yes	Yes	Yes	Yes	
30	1	LEV‐	No		96	No	No	Yes	No	Yes	
31	1	LEV‐	No		1	No	No	Yes	Yes	Yes	
32	1	LEV‐	Yes	Generalized	22	Yes	Yes	No		No	PANS related
33	1	LEV1	No		45	Yes	No	Yes	Yes	Yes	
34	1	LEV‐	No		18	Yes	No	Yes	Yes	Yes	
35	1	LEV2	Yes	Unknown	24	Yes	No	Yes	Yes	Yes	
36	1	LEV‐	No		Exact timing unknown	Yes	No	Yes	Yes	Yes	
37	1	LEV‐	No		18	No	No	Yes	No	Yes	
38	1	LEV‐	Yes	Generalized	12	Yes	No	No		No	PANS related
39	1	LEV1	No		24	Yes	No	Yes	No	Yes	
40	1	LEV‐	No		0.5	No	No	Yes	No	Yes	
41	1	LEV‐	Yes	Generalized	132	No	No	Yes	No	Yes	
42	1	LEV1	No		0.5	Yes	No	Yes	Yes	Yes	
43	2	LEV1	No		48	Yes	No	Yes	No	Yes	
44	1	LEV1	Yes	Generalized	132	Yes	No	Yes	No	Yes	
45	1	LEV‐	Yes	Generalized	36	Yes	Yes	No		No	PANS related
46	1	LEV‐	No		12	Yes	No	Yes	No	Yes	
47	1	LEV‐	Yes	Generalized	18	Yes	No	No		No	PANS related
48	1	LEV2	No		24	Yes	No	Yes	No	Yes	
49	3	LEV2	No		12	Yes	No	Yes	Yes	Yes	
50	1	LEV‐	Yes	Unknown	30	Yes	Yes	No		No	PANS related
51	1	LEV1	Yes	Unknown	0.5	Yes	No	Yes	Yes	Yes	
52	1	LEV‐	Yes	Generalized	40	Yes	No	Yes	No	No	PANS related
53	2	LEV1	No		72	Yes	No	Yes	No	Yes	
54	1	LEV‐	Yes	Generalized	12	Yes	No	Yes	Yes	Yes	
55	1	LEV2	No		48	Yes	No	Yes	Yes	Yes	
56	1	LEV1	Yes	Unknown	4	Yes	No	Yes	Yes	Yes	
57	1	LEV‐	No		18	No	No	Yes	No	Yes	
58	1	LEV‐	No		12	Yes	No	Yes	Yes	Yes	
59	1	LEV‐	No		8	Yes	No	Yes	No	Yes	

Abbreviations: ARC, ameroid ring constrictor; BLH, British shorthair; BSH, British shorthair; DLH, domestic longhair; DSH, domestic shorthair; EHPSS, extrahepatic portosystemic shunt; FE, female entire; FN, female neutered; IHPSS, intrahepatic portosystemic shunt; LD, left division; LEV, levetiracetam; ME, male entire; MN, male neutered; PA, portoazygos; PANS, postattenuation neurologic signs; PC, portocaval; TFB, thin film band.

### 3.2 Shunt morphology

Shunts were most commonly identified using abdominal ultrasound (*n* = 37), followed by computed tomography angiography (*n* = 27), and intraoperative mesenteric portovenography (*n* = 7). There were 55 (93.2%) EHPSS (51 portocaval and 4 portoazygos) and four left‐divisional (6.8%) IHPSS.

### 3.3 Preoperative neurologic signs

All cats were recorded as having preoperative neurologic signs. A total of 13 (22.0%) had a history of preoperative seizures. Preoperative neurologic signs other than seizures included lethargy (*n* = 40); ptyalism/drooling (*n* = 35); ataxia (*n* = 17); blindness (*n* = 12); obtundation (*n* = 12); abnormal behavior (*n* = 12); tremors (*n* = 11); and five or less of head pressing, vacant episodes, hyperesthesia, disorientation, circling, facial twitching, mydriasis, collapse, rolling, vocalization, hyperactivity, and pacing. Two (3.4%) cats had neurologic signs immediately preoperatively, which included lethargy and blindness in one cat and tremors in another.

### 3.4 Preoperative and immediate postoperative medical management

All (100.0%) cats received preoperative medical management. A total of 58 (98.3%) cats received immediate postoperative medical management (Table S1).

### 3.5 Pretreatment with levetiracetam and phenobarbital

A total of 27 (45.8%) cats received pretreatment with LEV, including 18 (30.5%) that received LEV1 (4 had preoperative seizures), and nine (15.3%) that received LEV2 (4 had preoperative seizures). Three (5.1%) cats received pretreatment with phenobarbital (2 mg/kg IV immediately preoperatively, with loading up to 12 mg/kg in 12 h postoperatively and continued orally every 12 h for 29 days postoperatively [*n* = 1]; 2 mg/kg orally every 12 h commencing 9–14 h preoperatively and continued IV/orally for ≥4 months postoperatively [*n* = 2]), alone or in combination with LEV.

### 3.6 Surgical details

Method of shunt attenuation included suture ligation in 28 (47.4%) cats, thin film banding in 21 (35.6%), and ameroid constrictor in 10 (16.9%). Overall, degree of acute intraoperative shunt attenuation was recorded as none in four (6.8%), partial in 42 (71.2%), and full/complete in 13 (22.0%). A total of 52 (88.1%) cats underwent one cPSS attenuation surgery, six (10.2%) underwent two cPSS attenuation surgeries, and one underwent three attenuation surgeries. A total of 19 (32.2%) cats underwent surgery prior to January 1, 2017, whereas the remaining 40 (67.8%) underwent surgery after that time.

### 3.7 PANS development and treatment of PANS

PANS occurred after a single cPSS surgery in 52 (88.1%) cats, after the first of two cPSS attenuation attempts in five (8.5%), after the second of two cPSS attenuation surgeries in one (1.7%), and after all three cPSS surgeries in a (1.7%) cat with preoperative seizures. Postattenuation neurologic signs commenced after a median (range) of 26 h (0.5–132) postoperatively (*n* = 58). Timing of PANS development was not recorded for one cat. Three cats developed PANS 24–48 h after discharge, and all returned for treatment. A total of 26 (44.1%) cats developed PAS (four had preoperative seizures), which included generalized PAS in 16 (61.5%), partial/focal in one (3.9%), and unknown in nine (34.6%) (Table 1). Other PANS included tremors (*n* = 23); blindness (*n* = 22); ataxia (*n* = 10); muscle twitching (*n* = 10); and obtundation (*n* = 9); and three or less of hyperexcitability, lethargy, abnormal behavior, head pressing, hyperactivity, head bobbing, circling, mydriasis, hyperesthesia, disorientation, and opisthotonos. A total of 47 (79.7%) cats received treatment of PANS (Tables 1 and S2), 10 (16.9%) cats received no treatment and for the remaining two (3.4%) cats, this information was not available. No cat was recorded as having experienced a severe postoperative complication during treatment of PANS.

### 3.8 Serum electrolytes, ammonia, and glucose concentrations

Serum electrolytes, ammonia, and glucose concentrations within 7 days postoperatively but prior to commencement of PANS and values obtained closest to PANS onset are presented in Tables S3 and S4. Heatmaps illustrating the percentage of cases without derangements in these variables, overall, and among survivors and non‐survivors, are presented in Tables 2 and 3.

**TABLE 2:** Heatmap illustrating the percentage of cases without derangements in closest serum electrolytes, ammonia, and glucose concentrations to PANS onset overall and among survivors and non‐survivors.

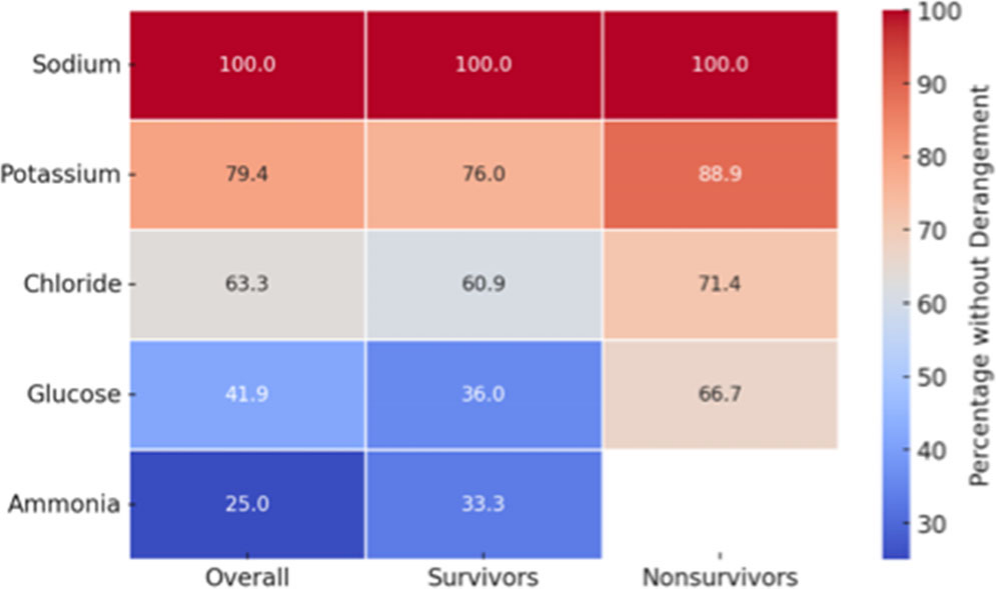

*Note*: The color gradient represents the proportion of patients without derangements, with higher values in red and lower values in blue. Reference intervals obtained from Silverstein and Hopper.^29^

Abbreviation: PANS, postattenuation neurologic signs.

**TABLE 3:** Heatmap illustrating the percentage of cases without derangements in most severe serum electrolytes, ammonia, and glucose concentrations within 7 days postoperatively but before PANS onset overall, and among survivors and non‐survivors.

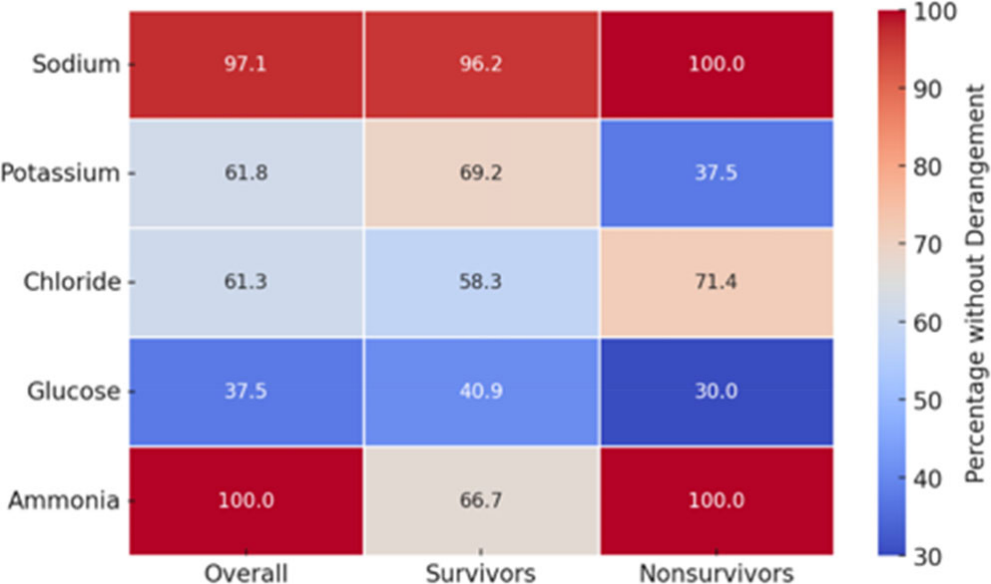

*Note*: The color gradient represents the proportion of patients without derangements, with higher values in red and lower values in blue. Reference intervals obtained from Silverstein and Hopper.^29^

### 3.9 Postoperative hospitalization and short‐term survival

A total of 47 (79.7%) cats survived to discharge after PANS occurrence (including all three cats that experienced PANS at home and returned for treatment). Postattenuation neurologic signs remained at discharge in 28 (59.6%) cats surviving to discharge. Median (range) duration of postoperative hospitalization for cats surviving to discharge was six (1–18) days. Median (range) time to death/euthanasia for cats not surviving to discharge was four (0.5–15) days. A total of 46 (78.0%) cats survived to 30 days. 13 (50.0%) of 26 cats that experienced PAS survived to 30 days, two of which had preoperative seizures. Of the 13 non‐surviving cats (2 had preoperative seizures), all experienced PAS, with 10 (76.9%) experiencing generalized PAS and three experiencing unknown seizure type. Nine (69.2%) were humanely euthanized due to PANS, three (23.1%) died due to uncontrolled generalized PAS, and one (7.7%) died due to postoperative hemorrhage. Of the 46 cats that did survive to 30 days (11 had preoperative seizures), 33 (71.7%) were recorded as not having experienced PAS, six (13.0%) experienced generalized PAS, one (2.2%) experienced focal seizures only, and in the remaining six, the type of PAS was unknown.

### 3.10 Prognostic factors for short‐term survival

Cats that experienced PAS had decreased odds of survival to 30 days (*p* = .005, OR: 0.015, 95% CI: <0.001–0.281) compared with those that experienced non‐seizure PANS (Table S1). Treatment of PANS with propofol was also associated with decreased odds of survival (*p* = .0008, OR: 0.112, 95% CI: 0.022–0.569) (Table S2). LEV group approached statistical significance (*p* = .06), with 20/33 (60.6%), 17/17 (100.0%) and 8/9 (88.9%) cats in groups LEV‐, LEV1 and LEV2, respectively, surviving to 30 days. Cats that underwent surgery in the second half of the study period did not have increased odds of survival to 30 days (*p* = .91). For those that experienced PAS, having a history of preoperative seizures was not associated with short‐term survival (50.0% vs. 50.0%, *p* > .99) (Table S1). Using point biserial correlation, bodyweight and age at the time of surgery were positively correlated (*r* = 0.46), whereas development of PAS was negatively correlated with survival to discharge (*r* = −0.57) and survival to 30 days (*r* = −0.60) (Table 4).

**TABLE 4:** Point biserial correlation illustrating the relationship between selected dichotomous (sex [male/female], presence of preoperative seizures [yes/no], shunt morphology [EHPSS/IHPSS], degree of cPSS attenuation [partial or none vs. full], LEV group [LEV2 or LEV‐vs. LEV1], developed PAS [yes vs. no], treatment of PANS [yes vs. no], treatment of PANS specifically with propofol [yes/no], survival to discharge [yes/no] and survival to 30 days [yes/no]) and continuous (age [months] and bodyweight [kg] at surgery, timing of PANS development [hours postoperatively]) variables.

	Age (months)	Sex	Weight (kg)	Preoperative seizures	Shunt morphology	Degree of attenuation	LEV group	Developed PAS	Timing developing PANS	Treatment of PANS	Treatment with propofol	Survival to discharge	Survival 30 days postoperatively
Age (months)	1												
Sex	−0.12299	1											
Weight (kg)	0.45565	−0.05392	1										
Preoperative seizures	0.09206	0.0858	0.05584	1									
Shunt morphology	−0.01397	−0.03226	0.01701	−0.0193	1								
Degree of attenuation	0.26567	−0.00301	0.20359	0.2107	−0.0193	1							
LEV group	−0.00966	−0.17542	−0.14929	0.04365	0.1645	0.04365	1						
Developed PAS	−0.06071	0.21741	−0.09826	−0.1424	0.1645	0.02234	−0.0807	1					
Timing developing PANS	−0.07042	−0.00556	0.07091	0.00633	−0.03304	0.14593	0.14824	0.13061	1				
Treatment of PANS	0.06117	−0.1149	−0.35215	0.03086	−0.12672	0.03086	0.28815	0.13061	−0.10847	1			
Treatment with propofol	−0.10247	0.16585	−0.2353	−0.21963	−0.08672	0.02112	−0.25241	0.35536	0.01303	0.18638	1		
Survival to discharge	0.15099	−0.1519	0.21331	0.16703	0.19872	−0.03616	0.30822	−0.56926	0.08748	−0.22556	−0.44233	1	
Survival 30 days postoperativ	0.09994	−0.17461	0.15553	0.08528	0.18196	−0.11204	0.32428	−0.59891	0.06804	−0.2382	−0.41079	0.95049	1

Abbreviations: EHPSS, extrahepatic portosystemic shunt; IHPSS, intrahepatic portosystemic shunt; LEV, levetiracetam; PANS, postattenuation neurologic signs; PAS, postattenuation seizures.

## 4 DISCUSSION

The main findings of this study were that (1) the majority (78.0%) of cats that experienced PANS survived to 30 days; however, the short‐term survival rate was lower for cats that experienced PAS (50.0%), and (2) prognostic factors negatively associated with short‐term survival included development of PAS and treatment of PANS with propofol. Therefore, we accept our first hypothesis but can only partially accept our second hypothesis.

The short‐term survival rate of PANS‐affected cats in our study was good, with 78.0% surviving to 30 days. Our results are in agreement with previous studies in which short‐term survival of PANS‐affected cats is reported.^5^, ^14^, ^18^ Within such studies,^5^, ^14^, ^18^ the 30‐day survival rate ranged from 66.7% to 100.0%. Just under half (47.4%) of the cats in our study received attenuation with suture ligation. This is most likely related to the inclusion of several cases from an institution whose current protocol is to attempt to achieve complete acute ligation, with partial attenuation with application of TFB performed in cases where this is not tolerated. Some historic cases will also have received staged suture ligation over two surgeries although this is no longer perfomed.^1^ The short‐term survival rate for cats that experienced PAS in our study was less favorable, with just 50.0% of such affected cats surviving to 30 days. This is in agreement with what has been published in the literature, with cats that experience milder PANS generally having a better prognosis for survival compared with those that experience more severe forms of PANS such as coma or seizures.^1^, ^5^, ^6^, ^7^, ^9^, ^12^, ^13^, ^14^, ^20^ This would appear intuitive as more severe forms of PANS would be more challenging to control, associated with greater treatment cost, more distressing for the owner to observe, and possibly associated with a greater risk of complications.^2^ A recent meta‐analysis that pooled data from previously published feline PANS cases found an overall mortality rate of 17.0% (95% CI: 8.8%–25.1%) for those affected by PANS and 37.2% (95% CI: 24.4%–49.9%) for cats that experienced PAS.^15^ The results of our study are slightly different in that the short‐term mortality rate (50.0%) was higher for PAS‐affected cats.

While it could be suggested that some of the neurologic signs observed within seven postoperatively in our study were simply a continuation of preoperatively observed neurologic signs, all 59 cats in our study received preoperative medical management and only two cats were recorded as having neurologic signs immediately preoperatively. We did not have electrolyte, glucose and ammonia concentrations around the time of PANS occurrence available for all cats and this is a limitation of our study. While some cats had abnormalities in these parameters, they were typically not very severe. There is evidence in dogs that PANS can progress despite treatment to correct hypoglycemia.^17^, ^21^ Furthermore, while hypocalcemia and hypokalemia have been identified in dogs affected by PAS, correction with intravenous supplementation did not always abolish seizures.^17^, ^22^ Thus, such electrolyte abnormalities may not have been the cause of PANS in these cases.^17^, ^21^, ^22^

Cats that experienced PAS in our study had reduced odds of survival to 30 days compared with those that experienced less severe PANS. The short‐term mortality rate of just over 20% for PANS‐affected cats in our study was likely directly related to the occurrence of generalized PAS, with just over 75% of non‐surviving PANS‐affected cats having developed generalized PAS, while for the other 25%, the type of seizure experienced was unknown. We also found that cats that received treatment with propofol had decreased odds of survival, which is likely explained by the fact that propofol was administered to the most severely affected cases and thus is a marker of severity of PANS. This observation corroborates findings of other studies involving dogs and cats affected by PANS.^1^, ^23^ In dogs, it has been shown that development of non‐seizure PANS and occurrence of focal PAS as opposed to generalized PAS are associated with better short‐term survival.^3^, ^19^, ^22^, ^23^, ^24^, ^25^, ^26^, ^27^ Milder PANS are likely easier to control, less distressing for an owner to endure, less expensive to treat, and less likely to result in euthanasia. Our study spanned a 13.5‐year period, and we hypothesized that cats that experienced PANS in the second half of the study period would have improved short‐term survival compared with those that experienced this complication in the first half of the study period. This hypothesis was based on the premise that, with greater awareness of this complication and its management combined with improvements in critical care medicine, the short‐term survival rate would be greater. This was not supported by our results and is in agreement with a previous study in dogs in which short‐term survival was not associated with study time frame.^3^ A significant limitation of assessment of this variable in veterinary surgery is that survival to 30 days is potentially influenced by several other factors including the client's willingness to pursue treatment, the experience of staff managing this complication (particularly relevant in a multi‐institutional study), cost of treatment, and managing clinician's perception of likelihood of neurologic recovery. The third part of our second hypothesis was based on the results of a previous study in dogs, in which those that experienced PAS that had a history of preoperative seizures had better short‐term survival than dogs without preoperative seizures.^3^ The reason for this was not entirely clear but was not supported by the results of our study. Finally, there was a trend toward cats that received pretreatment with LEV having improved survival to 30 days; however, this did not reach statistical significance. While the use of antiseizure medications such as LEV for the prevention of PANS remains controversial,^1^ it is possible that such pretreatment may result in improved short‐term survival if there is occurrence of PANS, but further studies are required to investigate this further.

In our study, we included cats that experienced PANS within the first 7 days postoperatively.^3^, ^28^ This differs from the definition of PANS described by Strickland and colleagues,^1^, ^23^ in which investigators defined PANS as any abnormal neurologic signs between surgery and discharge. While PANS typically occur within the first 96 h postoperatively,^1^, ^4^, ^5^, ^6^, ^7^, ^8^, ^9^, ^10^ we extended this to 7 days postoperatively to capture any cases of PANS that occurred after discharge.^3^, ^28^ Interestingly, three cats (5.1%) in our study developed onset of PANS after discharge.

We recognize several important limitations in our study. This was a retrospective study and therefore the accuracy of collected data depends on completeness of the medical records. For example, the classification of PAS as focal or generalized reflects what was recorded in the medical record. This was a multi‐institutional study involving multiple clinicians, with differences in experience recognizing and managing this complication. Several other non‐clinical factors may have influenced the 30‐day survival rate, including the willingness of the client to continue treatment, the cost of treatment across different institutions, the extent to which PANS were treated, and attending surgeon's perception of prognosis for neurologic recovery and whether it was worthwhile continuing treatment. Although the number of cats included in our study was larger than any other previously published study, the number was still relatively low. This precluded being able to run a multivariable logistic regression analysis, and therefore our results should be interpreted in light of this. While we assessed treatment with several antiseizure drugs as possible prognostic factors, treatment was not randomized but rather based on clinician preference, which was a limitation of our study, and some cats received combinations of drugs. Finally, because of the multicenter nature of the study, in cases where clinicopathologic variables were available, these variables were obtained from several different analyzers and therefore we had to use reference intervals from an emergency and critical care textbook.

In conclusion, most cats experiencing PANS survived up to 30 days. Cats that experience PAS and those that require treatment with propofol may have poorer short‐term survival.

## AUTHOR CONTRIBUTIONS

Otero Balda I, DVM, MSc: Data collection (1 case), data analysis and interpretation, manuscript preparation, approval of the final version of the manuscript. Selmic LE, BVetMed (Hons), MPH, DACVS (Small Animal), DECVS: Statistical analysis and interpretation, critical review of initial and final drafts and approval of the final version of the manuscript. Stamenova P, BVetMed, PGDip (VCP): Data collection (contribution of data related to 26 cases), critical review of initial and final drafts and approval of the final version of the manuscript. Simpson M, BVMS, MVetMed, DECVS: Data collection (contribution of data related to 26 cases), critical review of initial and final drafts and approval of the final version of the manuscript. Lipscomb VJ, MA, VetMB, DECVS: Data collection (contribution of data related to 26 cases), critical review of initial and final drafts and approval of the final version of the manuscript. Kummeling A, DVM, PhD, DECVS: Data collection (9 cases), critical review of initial and final drafts and approval of the final version of the manuscript. Devriendt N, DVM, PhD, DECVS: Data collection (contribution of data related to 6 cases), critical review of initial and final drafts and approval of the final version of the manuscript. de Rooster H, DVM, PhD, DECVS: Data collection (contribution of data related to 6 cases), critical review of initial and final drafts and approval of the final version of the manuscript. Grzywa KM, DVM: Data collection (contribution of data related to 6 cases), critical review of initial and final drafts and approval of the final version of the manuscript. Tivers MS, BVSc (Hons), PhD, DECVS: Data collection (contribution of data related to 6 cases), critical review of initial and final drafts and approval of the final version of the manuscript. Chanoit G, DEDV, MSc, PhD, DECVS, DACVS (Small Animal): Data collection (contribution of data related to 4 cases), critical review of initial and final drafts and approval of the final version of the manuscript. Maggiar A, DVM: Data collection (contribution of data related to 3 cases), critical review of initial and final drafts and approval of the final version of the manuscript. Billet JP, DECVS: Data collection (contribution of data related to 3 cases), critical review of initial and final drafts and approval of the final version of the manuscript. Soto Muñoz R, DVM: Data collection (2 cases), critical review of initial and final drafts and approval of the final version of the manuscript. Oramas A, DVM: Data collection (1 case), critical review of initial and final drafts and approval of the final version of the manuscript. Singh A, BSc, DVM, DVSc, DACVS (Small Animal): Data collection (1 case), critical review of initial and final drafts and approval of the final version of the manuscript. Mullins RA, MVB, DVMS, DECVS, PGDipUTL: Study conception and design, data analysis and interpretation, manuscript preparation, approval of the final version of the manuscript.

## CONFLICT OF INTEREST STATEMENT

The authors declare no conflicts of interest related to this study.

## Supporting information


**Data S1.** Supporting Information.
