# Supplementary material for: Prognostic factors for short‐term survival of cats that experienced postattenuation neurologic signs after surgical attenuation of single congenital portosystemic shunts
Source: Vet Surg. 2025 May 11;54(5):930–44. doi: 10.1111/vsu.14267 (PMC12282038; doi:10.1111/vsu.14267)
Supplement: Supplementary file 1 — Data S1. Supporting Information. [file VSU-54-930-s001.docx]

**Supplementary Tables**

**Supplemental Table 1:** Descriptive statistics and results of univariable regression analysis of variables investigated for possible associated with 30-days survival. Abbreviation: N/A, not applicable; NR, not run, EHPSS, extrahepatic portosystemic shunt; IHPSS, intrahepatic portosystemic shunt; LEV, levetiracetam; PAS, postattenuation seizures; PANS, postattenuation neurologic signs.

^a^Except for age, weight, duration of postoperative hospitalization for cats surviving to discharge, and time to death/euthanasia for cats not surviving to discharge.

| **Variable** | **Category^a^** | **n** | **%a** | **Survivors (n=46)** | **Nonsurvivors (n=13)** | ***P*-value** |
| --- | --- | --- | --- | --- | --- | --- |
| **Age** | Median (range), mo | 59 | 14 (4-55) | 14 (4-55) | 11.5 (4-33) | 0.45 |
| **Weight** | Median (weight), kg | 59 | 2.9 (0.9-5.4) | 2.9 (0.9-5.4) | 2.5 (0.9-4.1) | 0.23 |
| **Breed** | Domestic shorthair | 25 | 42.4 | 20 | 5 | 0.98 |
|  | British shorthair | 11 | 18.6 | 8 | 3 |  |
|  | Ragdoll | 11 | 18.6 | 8 | 3 |  |
|  | Other | 12 | 20.3 | 10 | 2 |  |
| **Sex** | Male | 41 | 69.5 | 30 | 11 | 0.19 |
|  | Female | 18 | 30.5 | 16 | 2 |  |
| **Neuter status** | Entire | 33 | 55.9 | 25 | 8 | 0.65 |
|  | Neutered | 26 | 44.1 | 21 | 5 |  |
| **Method of shunt identification** | Abdominal ultrasound | 37 | 62.7 | 29 | 8 | NR |
|  | Computed tomography angiography | 27 | 45.8 | 22 | 5 |  |
|  | Intraoperative mesenteric portovenography | 7 | 11.9 | 7 | 0 |  |
| **Shunt morphology** | EHPSS - Portocaval | 51 | 86.4 | 41 | 10 | 0.19 |
|  | EHPSS - Portoazygos | 4 | 6.8 | 3 | 1 |  |
|  | IHPSS - Left divisional | 4 | 6.8 | 2 | 2 |  |
| **Preoperative neurologic signs** | Yes | 59 | 100 | 46 | 13 | NR |
|  | No | 0 | 0 | 0 | 0 |  |
| **Neurologic signs immediately preoperatively** | Yes | 2 | 3.4 | 2 | 0 | 0.82 |
|  | No | 57 | 96.6 | 44 | 13 |  |
| **Presence of preoperative seizures** | Yes | 13 | 22 | 9 | 2 | 0.52 |
|  | No | 46 | 78 | 37 | 9 |  |
| **Preoperative medical management** | Yes | 59 | 100 | 46 | 13 | NR |
|  | No | 0 | 0 | 0 | 0 |  |
| **Preoperative antimicrobial for minimum of 1 week** | Yes | 38 | 64.4 | 31 | 7 | 0.37 |
|  | No | 21 | 35.6 | 15 | 6 |  |
| **Preoperative lactulose for minimum of 1 week** | Yes | 56 | 95.0 | 45 | 11 | 0.13 |
|  | No | 3 | 5.0 | 1 | 2 |  |
| **Preoperative protein-restricted/hypoallergenic diet for minimum of 1 week** | Yes | 57 | 96.6 | 42 | 13 | 0.83 |
|  | No | 2 | 3.4 | 2 | 0 |  |
| **Immediate postoperative medical management** | Yes | 58 | 98.3 | 46 | 12 | NR |
|  | No | 1 | 1.7 | 0 | 1 |  |
| **Antimicrobial immediately postoperatively** | Yes | 42 | 71.2 | 34 | 8 | NR |
|  | No | 17 | 28.8 | 12 | 5 |  |
| **Lactulose immediately postoperatively** | Yes | 57 | 96.6 | 45 | 12 | NR |
|  | No | 2 | 3.4 | 1 | 1 |  |
| **Protein-restricted/hypoallergenic diet immediately postoperatively** | Yes | 57 | 96.6 | 44 | 13 | NR |
|  | No | 2 | 3.4 | 2 | 0 |  |
| **Pre-treatment with** **LEV** | LEV- | 32 | 54.2 | 20 | 12 | 0.06 |
|  | Any LEV | 27 | 45.8 | 26 | 1 |  |
|  | LEV1 | 18 | 30.5 | 18 | 0 |  |
|  | LEV2 | 9 | 15.3 | 8 | 1 |  |
| **Pre-treatment with** **phenobarbital** | Yes | 3 | 5.1 | 2 | 1 | 0.54 |
|  | No | 56 | 94.9 | 44 | 12 |  |
| **Year of surgery** |  |  |  |  |  | 0.91 |
| **Surgery from 1st January 2017 onward** | Yes | 40 | 67.8 | 30 | 10 | 0.43 |
|  | No | 19 | 32.2 | 16 | 3 |  |
| **Number of shunt surgeries** | 1 | 52 | 88.1 | 39 | 13 | NR |
|  | 2 | 6 | 10.2 | 6 | 0 |  |
|  | 3 | 1 | 1.7 | 1 | 0 |  |
| **Method of shunt attenuation** | SL | 28 | 47.4 | 22 | 6 | 0.79 |
|  | TFB | 21 | 35.6 | 16 | 5 |  |
|  | ARC | 10 | 17.0 | 8 | 2 |  |
| **Degree of intraoperative attenuation** | None | 4 | 6.8 | 4 | 0 | 0.58 |
|  | Partial attenuation | 42 | 71.2 | 33 | 9 |  |
|  | Full attenuation | 13 | 22 | 9 | 4 |  |
| **Timing of onset of postattenuation neurologic signs** | Median (range), hours | 26 | (0.5-132) | 18 (0.5-132) | 24 (0.5-48) | NR |
| **Developed PAS** | Yes | 26 | 44.1 | 13 | 13 | 0.005 |
|  | No | 33 | 55.9 | 33 | 0 |  |
| **Type of PAS** | Generalized | 16 | 61.5 | 6 | 10 | 0.39 |
|  | Partial | 1 | 3.9 | 1 | 0 |  |
|  | Unknown | 9 | 34.6 | 6 | 3 |  |
| **Development of postoperative severe complications during  treatment of PANS** | Yes | 0 | 0 | 0 | 0 | NR |
|  | No | 59 | 100 | 46 | 13 |  |
| **Survival to discharge** | Yes | 47 | 79.7 | 46 | 1 | NR |
|  | No | 12 | 20.3 | 0 | 12 |  |
| **PANS remaining at discharge** | Yes | 28 | 59.6 |  |  | NR |
|  | No | 19 | 40.4 |  |  |  |
| **Duration of postoperative hospitalization for cats surviving to discharge** | Median (range), days |  |  | 6 (1-18) |  | NR |
| **Time to death/euthanasia for cats not surviving to discharge** | Median (range), days |  |  |  | 4 (0.5-15) | NR |
| **Survival to 30 days** | Yes | 46 | 78.0 | 46 | 13 | N/A |
|  | No | 13 | 22.0 | 0 | 0 |  |

**Supplemental Table 2:** Details of treatment of postattenuation neurologic signs. Abbreviation: CRI, constant rate infusion; PANS, postattenuation neurologic signs.

| **Variable** | **Category** | **n** | **%a** | **Survivors (n=46)** | **Nonsurvivors (n=13)** | ***P*-value** |
| --- | --- | --- | --- | --- | --- | --- |
| **Treatment of PANS** | Yes | 47 | 79.7 | 35 | 12 | 0.20 |
|  | No | 10 | 16.9 | 10 | 0 |  |
|  | Unknown | 2 | 3.4 | 1 | 1 |  |
| **Treatment with levetiracetam** | Yes | 38 | 86.4 | 28 | 10 | 0.23 |
|  | No | 6 | 13.6 | 6 | 0 |  |
| **Treatment with phenobarbital** | Yes | 37 | 84.1 | 29 | 8 | 0.72 |
|  | No | 7 | 15.9 | 3 | 4 |  |
| **Treatment with benzodiazepines** | Yes | 5 | 11.4 | 3 | 2 | 0.27 |
|  | No | 39 | 88.6 | 29 | 10 |  |
| **Treatment with propofol bolus(es) and/or CRI** | Yes | 7 | 15.9 | 2 | 5 | 0.0008 |
|  | No | 37 | 84.1 | 30 | 7 |  |
| **Treatment with gabapentin** | Yes | 1 | 2.2 | 1 | 0 | 0.94 |
|  | No | 43 | 97.7 | 31 | 12 |  |

**Supplemental Table 3:** Most severe serum electrolytes, ammonia, and glucose concentrations within seven days postoperatively but before PANS onset.

*Reference intervals obtained from Silverstein, D.C. and Hopper, K. (eds) Small Animal Critical Care Medicine 2nd edition. St Louis, MO: Elsevier Saunders; 2014.

| **Variable** | **Reference interval (RI)*** | **Overall** | **Survivors (n=46)** | **Nonsurvivors (n=13)** | ***P*-value** |  |
| --- | --- | --- | --- | --- | --- | --- |
| **Sodium** | 140.0-160.0 mmol/L | No derangements in 33/33 (100.0%) (mean [SD] 150.0 [4.3] mmol/L) | No derangements in 24/24 (100.0%) (mean [SD] 151.3 [3.3] mmol/L) | No derangements in 9/9 (100.0%) (mean [SD] 151.3 [3.3] mmol/L) | 0.73 |  |
| **Potassium** | 3.7-5.5 mmol/L | No derangements in 27/34 (79.4%) (median [range] 4.1 [3.7-5.4] mmol/L) | No derangements in 19/25 (76.0%) (mean [SD] 4.1 [0.4] mmol/L) | No derangements in 8/9 (88.9%)(mean [SD] 4.1 [0.5] mmol/L) | 0.98 |  |
|  |  | Hypokalemia in 7/34 (20.6%) (mean [SD] 3.5 [0.1] mmol/L) | Hypokalemia in 6/25 (24%) (mean [SD] 3.5 [0.1] mmol/L) | Hypokalemia in 1/9 (1.1%) (3.4 mmol/L) |  |  |
|  |  |  |  |  |  |  |
| **Chloride** | 119.0-132.0 mmol/L | No derangements in 19/30 (63.3%) (median [range] 121.5 [119.0-130.0] mmol/L) | No derangements in 14/23 (60.9%) (median [range] 121.0 [119.0-130.0] mmol/L) | No derangements in 5/7 (71.4%) (mean [SD] 122.0 [3.9] mmol/L) | 0.16 |  |
|  |  | Hypochloremia in 11/30 (36.7%) (median [range] 116.5 [113.0-118.0] mmol/L) | Hypochloremia in 9/23 (39.1%) (mean [SD] 116 [1.7] mmol/L) | Hypochloremia in 2/7 (28.5%) (both 117.0 mmol/L) |  |  |
| **Glucose** | 3.5-6.0 mmol/L | No derangements in 13/31 (41.9%) (mean [SD] 5.1 [0.4] mmol/L) | No derangements in 9/25 (36.0%) (mean [SD] 4.8 [0.6] mmol/L) | No derangements in 4/6 (66.7%) (mean [SD] 5.3 [0.3] mmol/L) | 0.22 |  |
|  |  | Hyperglycemia in 18/31 (58.1%) (median [range] 8.4 [6.2-15.3] mmol/L) | Hyperglycemia in 16/25 (64%) (median [range] 8.4 [6.2-15.3] mmol/L) | Hyperglycemia in 2/6 (33.3%) (6.1 and 9.6 mmol/L) |  |  |
| **Ammonia** | <50.0 µmol/L | No derangements in 1/4 (25.0%) (5.6 µmol/L) | No derangements in 1/3 (33.3%) (5.6 µmol/L) |  | 0.88 |  |
|  |  | Hyperammonemia in 3/4 (75.0%) (126.0, 146.0 and 223.0 µmol/L) | Hyperammonemia in 2/3 (66.7%) (126.0 and 146.0 µmol/L) |  |  |  |

**Supplemental Table 4:** Closest serum electrolytes, ammonia, and glucose concentrations to PANS onset.

*Reference intervals obtained from Silverstein, D.C. and Hopper, K. (eds) Small Animal Critical Care Medicine 2nd edition. St Louis, MO: Elsevier Saunders; 2014.

| **Variable** | **Reference interval (RI)*** | **Overall** | **Survivors (n=46)** | **Nonsurvivors (n=13)** | ***P*-value** |  |
| --- | --- | --- | --- | --- | --- | --- |
| **Sodium** | 140.0-160.0 mmol/L | No derangements in 33/34 (97.1%) (mean [SD] 147 [3.9] mmol/L) | No derangements in 25/26 (96.2%) (mean [SD] 146 [4.2] mmol/L) | No derangements in 8/8 (100%) (mean [SD] 150 [2.3] mmol/L) | 0.41 |  |
|  |  | Hypernatremia in 1/34 (2.9%) (165.0 mmol/L) | Hypernatremia in 1/26 (3.8%) (165.0 mmol/L) |  |  |  |
| **Potassium** | 3.7-5.5 mmol/L | No derangements in 21/34 (61.8%) (median [range] 4.0 [3.7-4.9] mmol/L) | No derangements in 18/26 (69.2%) (mean [SD] 4 [0.36] mmol/L) | No derangements in 3/8 (37.5%) (mean [SD] 3.9 [0.2] mmol/L) | 0.9 |  |
|  |  | Hypokalemia in 12/34 (35.3%) (mean [SD] 3.3 [0.2] mmol/L) | Hypokalemia in 8/26 (30.8%) (mean [SD] 3.4 [0.26] mmol/L) | Hypokalemia in 4/8 (50%) (mean [SD] 3.3 [0.1] mmol/L) |  |  |
|  |  | Hyperkalemia in 1/34 (2.9%) (5.7 mmol/L) |  | Hyperkalemia in 1 (12.5%) (5.7 mmol/L) |  |  |
| **Chloride** | 119.0-132.0 mmol/L | No derangements in 19/31 (61.3%) (median [range] 122.0 [120.0-132.0] mmol/L) | No derangements in 14/24 (58.3%) (median [range] 122 [120.0-132.0] mmol/L) | No derangements in 5/7 (71.4%) (mean [SD] 125 [2.2] mmol/L) | 0.44 |  |
|  |  | Hypochloremia in 12/31 (38.7%) (median [range] 116.5 [111.0-118.0] mmol/L) | Hypochloremia in 10/24 (41.7%) (median [range] 116.5 [111.0-118.0] mmol/L) | Hypochloremia in 2/7 (28.6%) (111.0 and 117.0 mmol/L) |  |  |
| **Glucose** | 3.5-6.0 mmol/L | No derangements in 12/32 (37.5%) (mean [SD] 4.9 [0.8] mmol/L) | No derangements in 9/22 (40.9%) (mean [SD] 5.1 [0.64] mmol/L) | No derangements in 3/10 (30.0%) (mean [SD] 3.9 [0.40] mmol/L) | 0.15 |  |
|  |  | Hypoglycemia in 1/32 (3.1%) (2.9 mmol/L) | Hypoglycemia in 1/22 (4.5%) (2.9 mmol/L) | Hyperglycemia in 7/10 (70.0%) (mean [SD] 9.7 [3.01] mmol/L) |  |  |
|  |  | Hyperglycemia in 19/32 (59.4%) (median [range] 8.8 [6.1-15.2] mmol/L) | Hyperglycemia in 12/22 (54.6%) (median [range] 8.4 [6.2-14.1] mmol/L) |  |  |  |
| **Ammonia** | <50.0 µmol/L | Hyperammonemia in 3/3 (100.0%) (113.0, 186.0 and 349.0 µmol/L) | Hyperammonemia in 2/3 (66.7%) (113.0 and 349.0 µmol/L) | Hyperammonemia in 1/1 (186.0 µmol/L) | 0.85 |  |
|  |  |  |  |  |  |  |
